# Regulatory and Mechanistic Actions of Glucocorticoids on T and Inflammatory Cells

**DOI:** 10.3389/fendo.2018.00235

**Published:** 2018-05-16

**Authors:** Ana C. Liberman, Maia L. Budziñski, Clara Sokn, Romina Paula Gobbini, Anja Steininger, Eduardo Arzt

**Affiliations:** ^1^Instituto de Investigación en Biomedicina de Buenos Aires (IBioBA) – CONICET – Partner Institute of the Max Planck Society, Buenos Aires, Argentina; ^2^Departamento de Fisiología y Biología Molecular y Celular, Facultad de Ciencias Exactas y Naturales, Universidad de Buenos Aires, Buenos Aires, Argentina

**Keywords:** glucocorticoids, inflammation, FKBP51, transactivation, transrepression

## Abstract

Glucocorticoids (GCs) play an important role in regulating the inflammatory and immune response and have been used since decades to treat various inflammatory and autoimmune disorders. Fine-tuning the glucocorticoid receptor (GR) activity is instrumental in the search for novel therapeutic strategies aimed to reduce pathological signaling and restoring homeostasis. Despite the primary anti-inflammatory actions of GCs, there are studies suggesting that under certain conditions GCs may also exert pro-inflammatory responses. For these reasons the understanding of the GR basic mechanisms of action on different immune cells in the periphery (e.g., macrophages, dendritic cells, neutrophils, and T cells) and in the brain (microglia) contexts, that we review in this chapter, is a continuous matter of interest and may reveal novel therapeutic targets for the treatment of immune and inflammatory response.

## Introduction

Living organisms must sustain a dynamic equilibrium in order to maintain homeostasis and survival which is constantly challenged by internal or external stressors. In order to appropriately cope with stressful stimuli, they have developed a highly conserved regulatory system. This neuroendocrine system consists mainly of the hypothalamic–pituitary–adrenal (HPA) axis and the autonomic nervous system. Glucocorticoids (GCs), are the end-product of the HPA axis, and play an important role in the maintenance of both resting and stress-related responses. If the stress response is dysregulated, homeostasis is altered and probably a wide range of adverse effects may appear on many vital physiological functions, such as growth, development, metabolism, reproduction, immune response, cognition, and behavior.

GCs act on almost all types of cells and in particular in the immune cells they have been shown to have powerful immunosuppressive and anti-inflammatory activities (1–5). As a result of their anti-inflammatory properties, GCs are widely used to help treat many different conditions, such as allergic, autoimmune, inflammatory, and hematological alterations. Interestingly, an accumulating body of evidence now strongly suggests that GCs can have both pro- and anti-inflammatory roles under specific conditions. The pro-inflammatory activity of GCs is most apparent in the central nervous system (CNS). These opposite effects work together in order to resolve cellular responses to inflammatory stimuli and also as a protective mechanism “priming” the immune cells to efficiently respond to the noxa or stressor and then restore homeostasis (6).

Upon peripheral or cerebral immune stimulation, the HPA axis is activated. When the immunogenic stress occurs in the brain, local inflammatory components activate the HPA axis. However, if the challenge takes place outside the brain, multiple pathways bring together stimulatory signals from the periphery to the HPA axis. Mounting evidence suggests that cytokine signals access to the brain through different pathways. These pathways mainly include: cytokines passing across the blood–brain barrier; by specific saturable transport molecules on the brain endothelium; activation of endothelial cells of brain capillaries that release second messengers within the brain parenchyma; transmission of cytokine signals *via* afferent nerve fibers and finally by peripherally activated monocytes that can enter into the brain (7–11). The induction of these different mechanisms modulates cytokine activity in the brain (12–14).

The accurate regulation of the HPA axis activity is critical, since GC imbalances can result in many different pathological conditions (13, 15). Long-term treatment with GCs may result in a plethora of harmful undesired side effects, such as diabetes, hypertension, growth retardation, dyslipidemia, osteoporosis, glaucoma, muscle atrophy, and is also related to many important behavioral alterations, among others (16, 17). Chronic exposure to GCs can also be associated with GC insensitivity, reducing the efficacy of the therapy (18). Also, alterations or deficits in the HPA axis response are tightly associated with a wide range of autoimmune and inflammatory diseases (19–24).

In this review, we will discuss the role of GCs on the immune and inflammatory cells in the periphery and also the physiological importance and mechanisms implicated in the apparent paradoxical functions of GCs in the brain in order to appropriately maintain a coordinated homeostatic response.

## The Glucocorticoid Receptor (GR)

**Figure 1:**
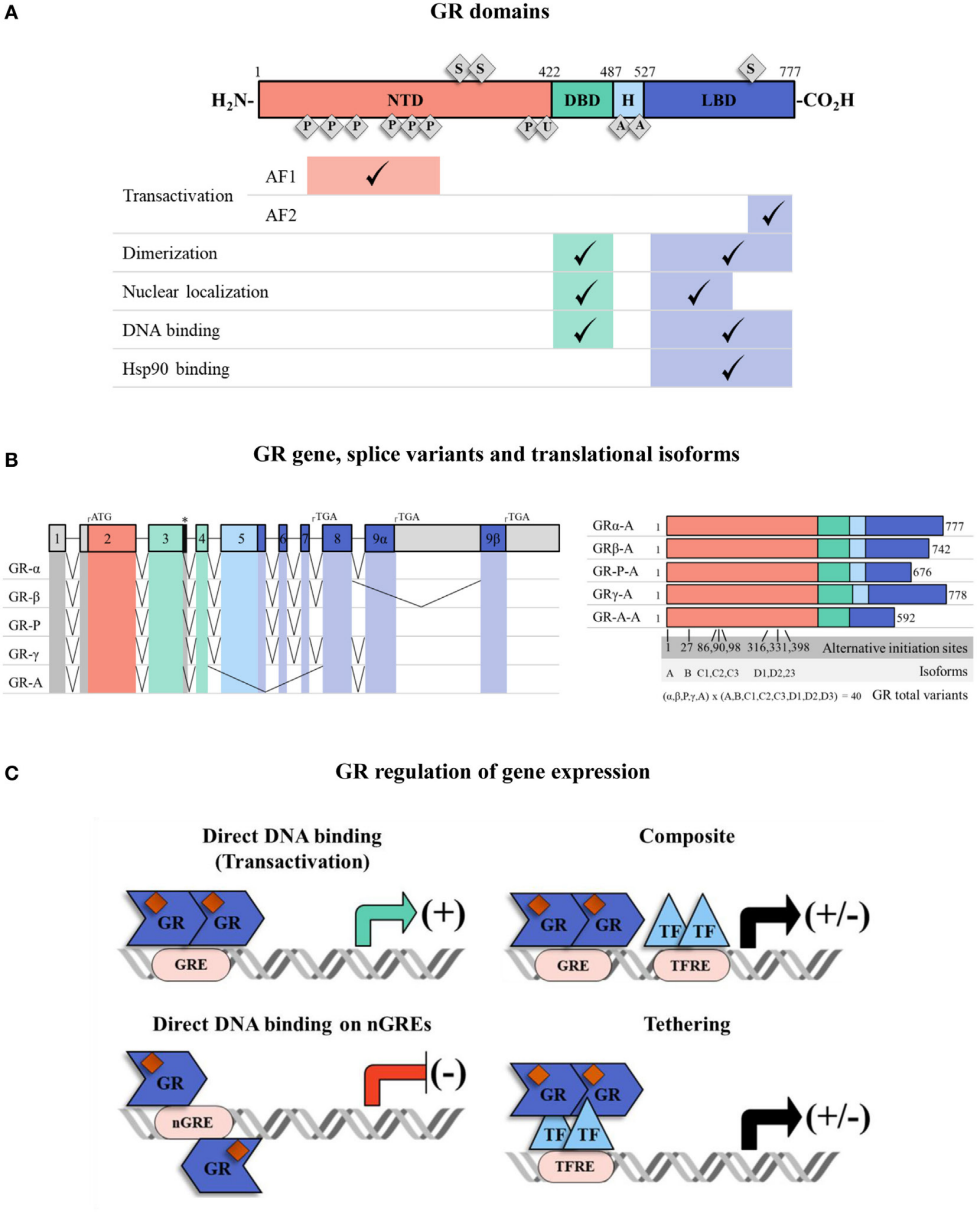
The glucocorticoid receptor (GR) structure, isoforms, and mechanisms of transcriptional regulation. **(A)** Full human GRα protein has an N-terminal domain (NTD), a DNA-binding domain (DBD), a ligand binding domain (LBD) and a hinge region (H) between DBD and LBD. They have different associated functions, e.g., transactivation, dimerization, nuclear localization, DNA binding, and heat-shock protein 90 binding. The receptor can be post-translationally modified by phosphorylation (P), ubiquitination (U), acetylation (A) and sumoylation (S). Regions associated with transactivation (activation function 1 and 2: AF1 and AF2) are shown. **(B)** The GR has various isoforms which result from alternative splicing and multiple transcriptional start sites at exon 2. The colors indicate NTD (red, exon 2), DBD (green, exons 3–4), H (light blue, exon 5) and LBD (dark blue, exon 5–9). The 5′ and 3′-untranslated regions are colored in gray. There are five patterns of alternative splicing that result in GR isoforms α, β, P, γ, A. Each of them has eight translational variants (A, B, C1, C2, C3, D1, D2, D3) depending on the transcriptional start site (“*” denotes an alternative splice donor site in the intron between exons 3 and 4). **(C)** The GR, carrying GC ligand, translocates to the nucleus and regulates gene expression. GR can directly activate/inactivate gene expression by interacting with GREs/nGREs, it can bind to GREs and modulate gene transcription by interacting with neighboring DNA-bound transcription factors (TFs) (composite mechanism) and it can act by attaching itself to DNA-bound TFs (tethering mechanism). Abbreviations: TF, transcription factor; GRE, glucocorticoid response element; nGRE, negative glucocorticoid response element; TFRE, transcription factor response element.

As a small lipophilic hormone, GCs can rapidly diffuse into cells and exert their main actions. These actions are elicited by the binding of GCs to their intracellular receptor, the GR. The GR is a hormone-activated transcription factor (TF) that belongs to the superfamily of nuclear hormone receptors (25). GR is a modular protein composed of three distinct regions with different functions (Figure 1A). The N-terminal domain (NTD) contains a transactivation domain called activation function 1 (AF1) that is responsible for the transcriptional activation and is implicated in the association with coregulators and the basal transcription machinery. The DNA-binding domain (DBD) is composed of two zinc fingers that have been shown to be important for GR homodimerization and DNA-binding specificity. The hinge region, which separates the DBD from the ligand binding domain (LBD), is a flexible linker structure which is implicated in allowing proper DNA binding, dimerization, and nuclear translocation of the receptor (26). The C-terminal LBD, contains the ligand binding site and a second transactivation domain (AF2) regulated by hormone binding (27). The AF2 transactivation domain is important for the interaction with co-chaperones, coregulators, and other TFs (28). The LBD also encompasses a dimer interface which is critical for GR function and the binding of the heat shock protein (Hsp) 90 (29). The DBD and LBD both contain nuclear localization signals, which are important for GR nuclear translocation. The DBD also contains the nuclear export signal sequence (NES) which targets it for export from the cell nucleus to the cytoplasm through the nuclear pore complex.

Some degree in the heterogeneity in GR proteins may result from alternative splicing (30) (Figure 1B). The specificity and sensitivity of different target tissues to GCs has been reported to be related to GR isoforms (30). The GRα is the predominant isoform, and it is the one that transduces GCs signaling in the cell (31). There are other four additional splice variants identified: GRβ, GRγ, GR-A, and GR-P. GRβ differs from GRα in the carboxy terminal sequence, rendering GRβ non-responsive to GCs (32, 33), with no transcription of target genes. Therefore, GRβ can be described as a dominant negative inhibitor of GRα activity. GRβ does not bind GC agonists, however, it does bind to the GR antagonist RU-486 (34). GRβ can inhibit GRα transcriptional activity by different molecular mechanisms including competition for glucocorticoid response elements (GRE), interference with the activity of coregulators, and formation of inactive dimers (35, 36). In most tissues, GRβ is expressed at very low levels. However, abundant GRβ expression has been described especially in some inflammatory cells, such as lymphocytes and macrophages, and have been related to GCs resistance in diseases such as asthma (37), rheumatoid arthritis (38), ulcerative colitis (39), systemic lupus erythematosus (40), and acute lymphoblastic leukemia and chronic lymphocytic leukemia (41, 42). Considering that GRβ can inhibit GRα activity, the modulation of GRα/GRβ expression ratios may be an interesting approach to regulate GC sensitivity (42, 43). In addition, eight alternative translation initiation sites increase the repertory of GR proteins to almost 40 distinct isoforms of GR protein (44) (Figure 1B).

At the cellular level, GC availability is also modulated by enzymes of the 11β-hydroxysteroid dehydrogenase (11β-HSD) family, mainly 11β-HSD1 and 11β-HSD2 which regulate the conversion of active cortisol into inactive cortisone. 11β-HSD1 favors the conversion of cortisol from cortisone, increasing local GC activity (45). In contrast, 11β-HSD2 catalyzes cortisol to cortisone, thereby reducing GC availability. Thus, the balance in the expression of these two enzymes in a given tissue or cell, regulates GC-mediated responses. In addition, some studies show that inflammatory cytokine signaling modulates the relative expression of 11β-HSD genes, favoring 11β-HSD1 and inhibiting 11β-HSD2 (46, 47), adding another level of regulation of GC activity.

Another important level for fine-tuning the cellular response to GCs in different environmental situations is the modulation of GR activity by posttranslational modifications (PTMs). These PTMs include phosphorylation, acetylation, ubiquitination, and sumoylation, which may accurately regulate GR activity in response to diverse external stimuli (48) (Figure 1A). In particular, SUMO conjugation has been extensively described to modulate GR transcriptional activity (49–52). GR contains three consensus sumoylation sites. Two sumoylation sites located at the NTD have been demonstrated to be part of the synergy control (SC) motif sequence (50). The SC motifs consist of short regulatory sequences which are important for inhibiting the synergistic transactivation. SUMO conjugation to the two NTD sumoylation sites is responsible for the functional effect of the SC motifs and thereby they inhibit GR activity (50, 53) (Figure 1A). It has also been demonstrated that in the presence of the sumoylation enhancer, RSUME (54), a SUMO peptide is conjugated to the third sumoylation site located in the LBD of the GR. Sumoylation in the LBD may be important for inducing GR-mediated transcriptional regulation during stress adaptation (55) (Figure 1A). A genome-wide analysis of GR sumoylation impact on gene expression, showed that genes differentially regulated by this PTM are mostly related to proliferation and apoptosis pathways and also strongly suggests that sumoylation can regulate genome-wide chromatin occupancy of the GR (56). Also, GR SUMO conjugation is influenced by other PTMs such as phosphorylation in order to fine-tune GR transcriptional activity in a target gene-specific manner (57). Important coregulators of the GR are also modified by SUMO conjugation, such as Hsp90, GRIP1, and also FKBP51, further regulating GR activity (58–62). Therefore, PTMs that impact on the GR but also on key molecules that fine-tune its activity, helps to understand the complexity of GR-mediated regulation of its target gene expression (2, 48).

## GCs Anti-Inflammatory Actions

The GR forms complex with chaperone molecules, such as Hsp90 and 70, and immunophilins, such as FKBP51, FKBP52, Cyp44, and PP5 (63). FKBP51 binds to the unbound GR and reduces GR activity mainly by reducing GR hormone binding and its nuclear translocation. Therefore, FKBP51 is considered as an inhibitor of GR transcriptional activity. Upon ligand binding, the GR exchanges FKBP51 for FKBP52, which is able to interact with the dynein motor protein, facilitating GR translocation to the nucleus (64). Interestingly, FKBP51 overexpression has been associated with GC resistance in autoimmune diseases. FKBP51 expression was found to be enhanced in sputum samples from patients with chronic obstructive pulmonary disease (65). Moreover, in a genome-wide profiling focused on the identification of epithelial gene markers of asthmatic patients and response to corticosteroids, GC treatment was found to induce FKBP51 expression, which in turn was associated with a poor response to corticosteroids, suggesting a role of FKBP51 in GC resistance (66, 67). Also, enhanced expression of FKBP51 has been found in bone marrow cells in patients with rheumatoid arthritis (68). Evidence also suggests that FKBP51 modulates NFĸB-dependent gene expression, with possible implications for various inflammatory and immune pathways (69–73). Considering that GR is a key modulator of immune and inflammatory responses, FKBP51 dysregulation may provide the basis for a role of FKBP51 in these processes (66). Moreover, FKBP51 has recently been shown to be a target of SUMO conjugation and that sumoylation of FKBP51 is necessary for its association to Hsp90 and modulates FKBP51-mediated inhibition of GR activity in neuronal cells (58). In the brain, FKBP51 has been shown to be important for the development of psychiatric diseases and the response to antidepressant treatment, suggesting that regulation of FKBP51 activity might be an interesting approach for modulating GR outcome in the stress response and also in the inflammatory context (74–76).

Once in the nucleus, the activated GR can regulate gene expression by different mechanisms known as genomic effects (Figure 1C) (27). The genomic mechanism involves changes in the levels of specific genes: binding of GR to GREs in the promoters of its target genes and activation of transcription (transactivation); DNA binding of the GR with other TFs to “composite” elements which contain a GRE and an overlapping response element of another TF (binding can lead to gene activation or repression); or binding of the GR to a TF (e.g., NFĸB; or AP1) by means of a “tethering” mechanism without contacting DNA, to influence the activity of the TF (this mechanism is considered to be the prevailing mechanism for transrepression) (2, 77, 78). Furthermore, GR-mediated transcriptional repression can be exerted *via* GR binding to a negative GRE (nGRE) (79). Binding to these nGRE prevent receptor dimerization through a strong negative cooperativity and alters the conformation of GR residues that are critical for transcriptional activation so that negative regulation is accomplished (80). A growing body of evidence shows that GC can also mediate non-genomic actions that do not require protein synthesis and are implicated in rapid cellular responses. For example, in the cytoplasm the activated GR can acutely interact with signaling pathways, such as PI3K, JNK, 14-3-3 proteins, and components of the T cell receptor signaling complex (81), modulating pro-inflammatory gene expression. In thymocytes, the activated GR can translocate to mitochondria and induce a rapid apoptotic response (82). In addition, membrane-bound GR on monocytes was reported to mediate non-genomic effects (82). On the other hand, binding of GCs to GR can modify the recruitment of different factors such as the multiprotein chaperone complex that participate in many signaling pathways, modifying secondary signaling cascades and, therefore, may further regulate the immune response (78, 83). GCs may also exert anti-inflammatory responses by direct negative interaction with components of the MAPK pathway, such as ERK, c-Jun NH2-terminal kinases (JNK), and p38 isoforms (p38) regulating their activity (84). Further studies are required to clarify the implications of non-genomic GC-mediated activity in the immune and inflammatory context.

It has been shown that several of the undesirable metabolic side effects associated with chronic GC treatment are mediated *via* transactivation. However the anti-inflammatory effects of GCs are mainly mediated *via* the transrepression elicited by a monomeric GR with the activity of TFs, such as NFĸB and AP1 (1–3, 85). These TFs are involved in the activation of pro-inflammatory and immunoregulatory genes, such as inflammatory cytokines, cytokine receptors, adhesion molecules, and chemotactic proteins that play a key role for the coordination of the inflammatory response (1, 86–88). The first example of the transrepressive mechanism was the inhibitory interaction described between GR and AP1 (89), which results in the inhibition of IL2 expression (90). NFĸB is present in almost all immune cells and regulates the expression of inflammatory cytokines. Thus, inhibition of NFĸB activity is an important feature for GR-mediated anti-inflammatory activity (85, 91). It also inhibits NFAT-dependent IL2 transcription (92). The main mechanism of the GR action over these TFs is *via* transrepression: the activated GR acts by binding proximal to the NFĸB or AP1-binding site and interacts with these TFs inhibiting gene expression (93). The transrepression mechanism is not restricted to these TFs, but has expanded including among others, CREB, STAT, and T-bet (1–3, 94).

Alterations in chromatin structure have been reported to be important for regulating GC actions. The GR can differentially interact with proteins that have histone acetyltransferase (HAT) activity, but also with histone deacetylases and kinases that can influence the chromatin environment modifying chromatin accessibility and further regulating immune and inflammatory gene expression (3). In addition, chromatin accessibility has been reported to pre-determine GR binding patterns and, therefore, is critical for cell-specific outcome, providing new molecular basis for the tissue selectivity (95, 96). By all these different mechanisms, GCs regulate important functions, not only in the periphery but also in the brain.

Synthetic analogs of GC are often employed in the clinic in the therapy of allergic, inflammatory, and autoimmune disorders (97–99). It is generally accepted that GR-mediated transrepression holds the beneficial anti-inflammatory action, whereas their side effects are due mainly to the direct binding of GR to GREs as depicted before (98–100). However, transactivation is also necessary for the induction of several anti-inflammatory genes, such as MAP kinase phosphatase 1 (101), glucocorticoid-induced leucine zipper (102), and inhibitor kappa B-alpha (IĸBα) (85). Therefore, the ideal GC analogs should be those that have high repressive activity against inflammatory mediators, but low transactivation activity, causing minimal side effects. Several steroidal and nonsteroidal ligands have been reported to have this dissociated function between transactivation and transrepressive mechanisms (97–99, 103). These compounds were shown to repress the activity of key inflammatory and immune TFs *in vivo* (104–107). However, GCs can induce gene expression not only by binding to GRE, but also in combination with other TFs and also by binding to promoter regions in a mechanism that does not involve GR dimerization or DNA interaction; therefore, unexpected secondary side effects might appear (78).

GCs may exert acute anti-inflammatory effects through the release of annexin-A1 (ANXA1) (108). Originally, this protein was suggested to have anti-inflammatory actions because it was described to inhibit phospholipase A2 (109). However, ANXA1 has been reported to regulate different cellular processes, such as migration, growth, differentiation, apoptosis, membrane fusion during exocytosis, lipid metabolism, and cytokine expression. Importantly, in the HPA axis, ANXA1 has been reported to play a critical role in the negative feedback exerted by GCs, therefore, affecting hypothalamic-releasing hormones secretion possibly *via* non-genomic mechanisms (110).

## GCs Activity on Peripheral Immune Cells

**Figure 2:**
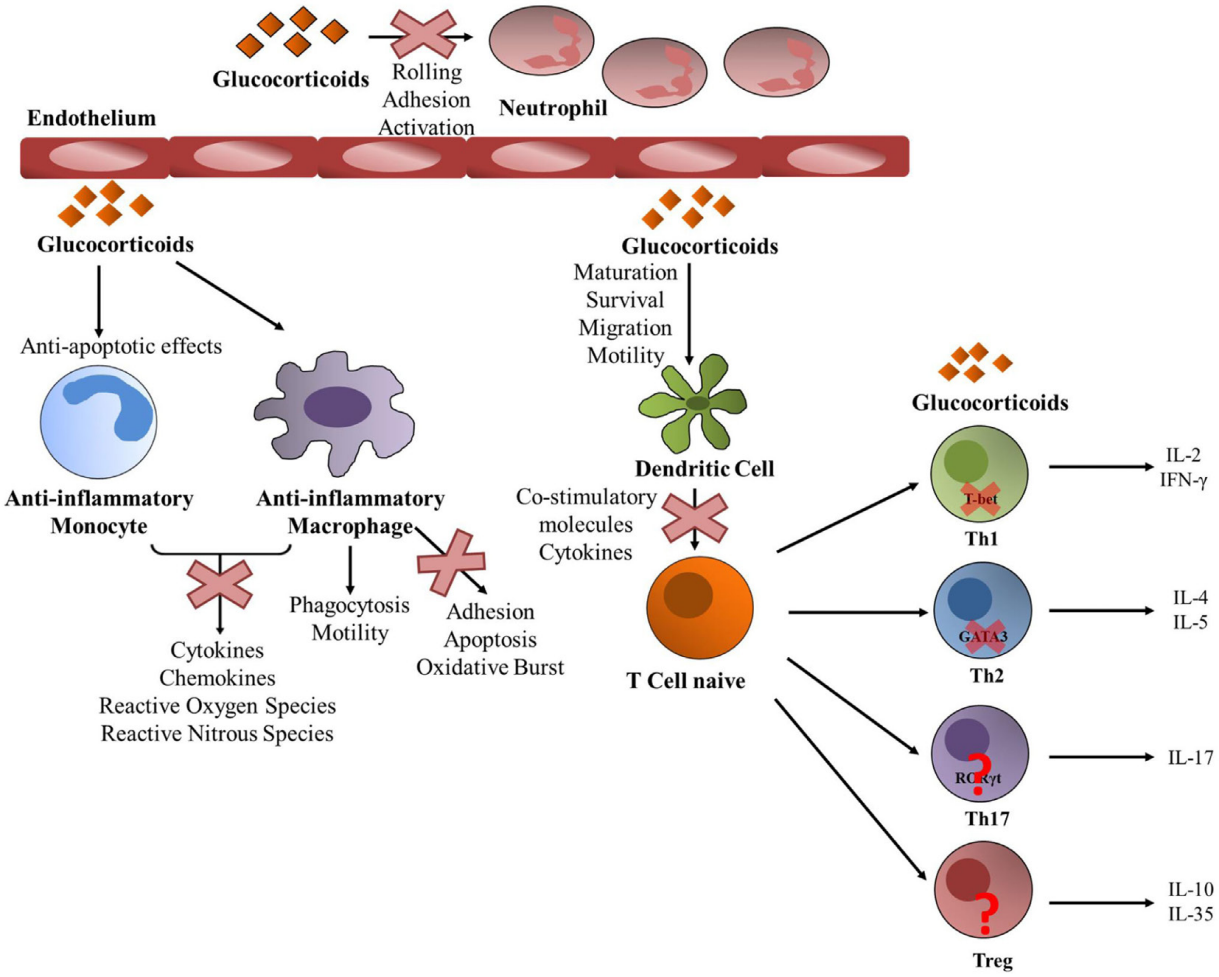
Glucocorticoid (GC) activity on periphery immune cells. GCs act upon almost every immune cell type. GCs promote an anti-inflammatory state on both monocytes and macrophages. GCs prevent monocytes into entering apoptosis and inhibit the liberation of pro-inflammatory mediators by both types of cells. Particularly in macrophages, GCs promote phagocytosis and motility, while they inhibit adhesion, apoptosis and oxidative burst. They also act upon neutrophils function by inhibiting rolling, adhesion and activation. GCs act toward dendritic cells by promoting their maturation, survival, migration and motility, and at the same time GCs inhibit their ability to activate T cells by suppressing the production of pro-inflammatory molecules. A naïve helper T (Th) cell can differentiate into different Th lineages and GCs exert different actions. They act upon Th1 by decreasing T-bet transcriptional activity and suppressing the production of pro-inflammatory molecules such as IL-2 and IFNγ. They also suppress GATA3 activity in Th2 cells inhibiting the expression of IL-4 and IL-5. The action of GCs toward Th17 and regulatory T cells is not yet well understood.

GCs mediate immunosuppressive functions by acting on almost all types of immune cells (Figure 2). GCs can regulate the phenotype, survival, and functions of monocytes and macrophages which have crucial roles in tissue homeostasis and innate immunity. GCs exhibit anti-apoptotic effects promoting the survival of anti-inflammatory macrophages (111). The intrinsic molecular mechanism involves a prolonged induction of the extracellular signal-regulated kinase/MAPK (ERK/MAPK) pathway resulting in inhibition of caspase activities and expression of anti-apoptotic genes (111). GCs can also improve the phagocytic activity of these cells and stimulate the clearance of harmful elements, such as neutrophil clearance (112–114). GCs also suppress immunostimulatory functions of these cells and inhibit the release of various pro-inflammatory mediators, such as cytokines, chemokines, and reactive oxygen through different mechanisms (115, 116). Functional clustering of GC-regulated genes by human anti-inflammatory macrophages by microarray technology indicated induction of phagocytosis and motility as well as repression of adhesion, apoptosis, and oxidative burst (117, 118).

GCs can regulate the maturation, survival, and migration toward the lymph nodes and motility of dendritic cells (DCs), and also inhibit their immunogenic functions (Figure 2). GCs were shown to reduce the ability of DCs to stimulate T cells by inhibiting the upregulation of co-stimulatory molecules and cytokines, such as IL6, IL12, and TNFα and by inducing the tolerance-inducing transcription factor GILZ (119–125). The distinct actions exerted by GCs in immature and mature DCs are due to differential expression of GR translational isoforms (126).

GCs are important modulators of neutrophilia (Figure 2). Leukocyte extravasation is the movement of leukocytes out of the circulation and toward the site of tissue damage or infection. Rolling, adhesion, activation, and transmigration are necessary to arrive to the damaged tissue. GCs can modulate each of these steps. Rolling and adhesion is mediated by the interaction of the leukocyte integrins with the endothelial counterparts, which are inhibited by GCs (127–129). Also, GCs increase the number of circulating neutrophils in the blood stream by favoring their egress from the bone marrow and also inhibiting their migration to inflammatory sites by hindering the expression of adhesion molecules (32, 129, 130).

GCs exert distinct immunomodulatory actions on T cells (Figure 2). GCs decrease the number of circulating T cells by favoring their migration back to the bone marrow and secondary lymphoid tissues or through the induction of chemokine receptors, adhesion molecules, and matrix metalloproteinases (131, 132). The steroid hormone also favors T cells apoptosis. GC-induced apoptosis of T cells requires the dimerization of the GR (133) and is mediated *via* the induction of Puma and Bim expression (134–137). The relative expression of distinct GR isoforms increases the susceptibility of T cells to GC-induced cell death (138). Helper T (Th) cells are important players of the adaptive immunity (1). Upon antigen stimulation, naive Th cells can differentiate into different subsets: Th1, Th2, Th17, or regulatory T (Tregs) cells among others, each with specific effector functions. Th1 cells express the lineage-specific TF T-bet and STAT4 and release pro-inflammatory cytokines, such as IFNγ and IL2 (139). Th1 cells help in the activation of effector T cells, natural killer (NK) cells, and macrophages at the site of infection, promote effective immune responses against intracellular pathogens and are also implicated in autoimmune pathologies. Th2 lymphocytes selectively express the TF GATA3 and are characterized by the expression of IL5, IL4, IL10, and IL13 and are important for the proper eradication of extracellular pathogens (140). Also, Th2 cells activate B cells to produce antibodies and play a triggering role in the activation/recruitment of eosinophils and mast cells in allergic responses. IL17-producing Th17 cells selectively express RORγt and also RORα (141, 142). Th17 cells play an important role in autoimmune diseases and in host defense against infection. Treg cells mainly express the TF Foxp3 and inhibit effector T-cell differentiation and proliferation and suppress autoimmune and allergic responses (143). GCs inhibit the expression of many T cell cytokines (1) and can produce a shift from Th1-mediated cellular immunity to mediating humoral Th2 responses at physiological doses or chronic treatment (144). Upon acute treatment with GCs, they inhibit the synthesis of Th1 cytokines like IL2 and IFNγ and reduce STAT4 activity (145) and also reduce Th2 cytokines expression (146). The molecular mechanism by which GCs inhibit Th1 responses involves the reduction of T-bet transcriptional activity by the inhibitory interaction between GR and T-bet that results in diminished binding of T-bet to DNA (94) (Figure 2). Also GCs where shown to reduce mRNA and protein levels of T-bet (94). The activity of the Th2-specific TF GATA3 is also suppressed by GCs *via* two main mechanisms: first by GR-mediated inhibition of GATA3 translocation into the nucleus and second by the inhibition of GATA3 phosphorylation by GC-induced MKP1 expression (147, 148) (Figure 2). Furthermore, STAT6 activity also involved in Th2 differentiation is inhibited by GCs (149). How GCs modulate Th17-mediated responses has not been extensively studied, and the importance of Th17 modulation by GCs for the suppression of allergic or autoimmune diseases remains unclear (150). In rheumatoid arthritis, GC treatment diminished IL17 levels (151). In addition, in rat lymphocytes methylprednisolone inhibited IL17 expression due to the inhibition of RORγt expression (152) (Figure 2). However, several studies strongly suggest that GC resistance is associated with a pathogenic inflammatory Th17 phenotype that is refractory to GCs (150, 153, 154). Recently, a gene-expression profiling to characterize the steroid-resistant phenotype showed that Th17 cells have restricted genome-wide responses to GCs and that they are refractory to GC inhibition at this level. In addition, Th17 cells were sensitive to suppression with the calcineurin inhibitor, cyclosporine A, suggesting that the clinical efficacy of cyclosporine A in the treatment of steroid resistance may be due to its selective inhibition of Th17 cells (155). Another interesting study has shown that Th17 cells are insensitive to GC-induced apoptosis and had high levels of BCL-2, knockdown of which sensitized Th17 cells to GC-induced cell death (156). Also, lung Th17 development in the murine severe asthma model was enhanced by GCs, supporting a role of Th17 cells in GC-refractory inflammatory conditions such as asthma (157).

In contrast to the inhibitory effect of GCs on pro-inflammatory effector T cells, it has been shown that Treg cells, which are key suppressors of T cell-dependent immune responses, are enhanced upon dexamethasone treatment by being more resistant to GC-induced cell death (158) (Figure 2). Also, GCs where shown to amplify IL2-dependent expansion of Treg cells and to enhance their capacity to reduce experimental autoimmune encephalomyelitis (EAE) in mice (159). In addition, GCs increase the percentage of Treg cells that express Foxp3 in patients with multiple sclerosis (160). *In vivo*, T cell-specific targeted GR deletion in pregnant animals undergoing EAE, resulted in a reduction of Treg population and a loss of pregnancy-induced protection, suggesting that steroid hormones can shift the immunological balance in favor of Tregs *via* differential engagement of the GR in T cells (161). However, others have found that GC treatment suppresses the expression of Foxp3 Tregs in an EAE model (162) and also in lungs of allergic mice (163).

In addition to their well-studied anti-inflammatory and immunosuppressive activity, an increasing body of evidence has revealed situations in which GCs have the opposite effect. This has been shown to depend on the dose, timing, duration of exposure, and cell population or tissue analyzed (164). The paradoxical pro-inflammatory role of GCs is mostly evident in the brain, where accumulating evidence show that GCs elicit different immune responses depending on the affected brain regions.

## GCs Actions in the Brain

There is a significant body of evidence indicating that GCs can suppress the innate immunity in the brain after a peripheral or cerebral challenge (23). In this way, in adrenalectomized mice, there is an induction in the levels of pro-inflammatory cytokines in the brain following LPS injection (165–168). Studies also demonstrated that GCs inhibit the release of pro-inflammatory mediators in microglial cells treated with LPS (169, 170). Experiments performed *in vivo* support these findings by revealing that dexamethasone causes a strong reduction in LPS induction of NFĸB expression in the brain (171). In addition, COX inhibitors were demonstrated to increase the expression of pro-inflammatory genes in the brain during systemic inflammation by reducing the activation of the HPA axis and the release of GCs (172, 173). This same effect took place when the GR antagonist RU486 was administrated (172, 173). Also, systemic inflammation, through the increase in circulating GCs, has been reported to have the ability to prevent the cerebral innate immune response induced by intraparenchymal endotoxin injection (174). Mice treated with the GR antagonist RU486 before intracerebral LPS administration showed an increase in the pro-inflammatory response, which in turn induced neuronal death. These findings suggest that GCs are important for protecting the brain during innate immunity (175, 176). Interestingly, when mice lacking GR in microglia were challenged with an intracerebral administration of LPS, the activation of the toll-like receptor 4 signaling pathway induced cellular lesion, and also neuronal and axonal damage (177). In addition, microglial cell cultures have reduced motility and increased amoeboid morphology in the absence of GR expression. This study strongly suggests that microglial GR is the principal mediator preventing neuronal degeneration triggered by LPS and that it also contributes to the protection of other cell types (177), having an important role in promoting neuronal survival.

**Figure 3:**
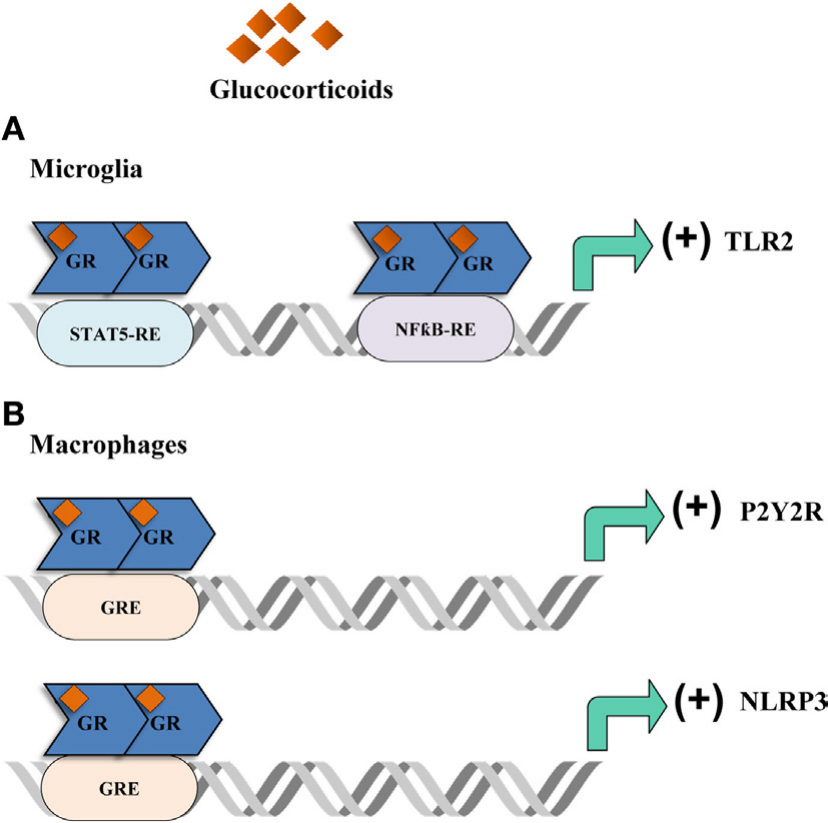
Glucocorticoids (GCs) actions in the brain. Acute stressors promote an inflammatory phenotype in the brain. **(A)** In the microglia, GCs bind to the glucocorticoid receptor (GR) which then promotes the translation of the toll-like receptor 2 (TLR2) by interacting with STAT5 and NFƙB response elements. TLR2 then exerts a pro-inflammatory response by promoting the production of inflammatory cytokines. **(B)** In macrophages, GCs promote the expression of the purinergic receptor P2Y2R which then produces IL-6 in response to ATP. Moreover, GCs enhance the expression of NLRP3 which in turn promotes the production of pro-inflammatory cytokines.

The majority of GC pro-inflammatory activity has been described in animal models of acute or chronic stress which occurred previous to peripheral or cerebral immune challenges. For instance, acute stressors were reported to induce the expression of pro-inflammatory cytokines in specific brain regions, such as the hippocampus, following LPS peripheral challenge (178–180). GCs were also found to upregulate microglial activation markers including the toll-like receptor 2 pro-inflammatory pathway (178, 181) (Figure 3A). It was also shown that chronic unpredictable stress was able to potentiate LPS-mediated activation of NFĸB activity in the frontal cortex and hippocampus *via* GC production (182). Also, chronically stressed animals that were injected with LPS in the prefrontal cortex or the hippocampus, exhibited microglia activation, an increase in pro-inflammatory mediators and loss of astroglia and neurons. These effects were reduced with RU486 administration (183, 184). The prefrontal cortex is important in many brain functions and is a target for neurodegenerative diseases. It has been reported that in this brain region, TNFα expression and activation of MAPK signaling pathway is upregulated by chronic stress after intracortical LPS injection in a GR-dependent manner suggesting a synergistic effect between inflammation and stress. This fact could ultimately explain the relationship described between stress and some neurodegenerative pathologies (183, 184). In order to investigate if stress-induced GCs is responsible for the response of brain immune cells to pro-inflammatory stimuli, animals were acutely stressed and 24 h later hippocampal microglia were challenged with LPS *ex vivo*. Treatment *in vivo* with RU486 and adrenalectomized inhibited the microglial pro-inflammatory response, indicating that stress-induced GCs are able to sensitize the microglial pro-inflammatory function (185, 186). Therefore, stress may act “priming” central innate immunity to a subsequent immune challenge by making the neuroimmune context more responsive to inflammation, also favoring GC insensitivity or reducing the HPA response (187). In addition, acute restraint stress, inescapable tail shock and other stressors induce many inflammatory mediators, reduce immunoregulatory proteins and trigger microglia activation and proliferation (188–193). In addition, GCs have been reported to increase the expression of the purinergic receptor P2Y2R (Figure 3B) which promotes the secretion of inflammatory mediators in response to ATP (194). Recent data also indicate that GCs induce the expression of NLRP3 (NLRP3: nucleotide-binding domain, leucine-rich-containing family, pyrin domain-containing 3) in macrophages, which is a critical component of the inflammasome (Figure 3B). The GC-dependent induction of NLRP3 sensitizes the cells to extracellular ATP and significantly enhances the ATP-mediated release of pro-inflammatory molecules. This effect was specific for GCs and dependent on the GR and suggests that GCs sensitize the initial inflammatory response in the context of acute cellular damage or death (32). In addition, GCs and TNFα were shown to coregulate immune gene expression when combined (195). These results suggest that the final outcome of GCs pro- or anti-inflammatory activity depends on the activation state and signaling context. GCs are also able to modulate the inflammatory response to LPS in different ways according to the brain region (180, 182). For example, GR activation during chronic stress increases LPS-induced NFκB activation and TNFα, IL1β, and iNOS expression in the hippocampus and frontal cortex, but exhibits contrary effects in the hypothalamus (182). It is important to keep in mind that a pro-inflammatory context does not necessarily mean that damage will take place. Timing is a key parameter that will determine the final outcome of the inflammatory response. While exaggerated inflammation can favor neuronal dysfunction and cell death, pro-inflammatory mediators may at first induce the removal of the pathogen, the recruitment of immune cells and initiate tissue remodeling in order to appropriately cope with the pathogen and therefore, restoring homeostasis.

## Conclusion

GCs are widely used in the clinic to control not only peripheral, but also CNS inflammatory response. However, the prolonged administration of this steroid hormone is often ineffective and can even worsen the outcome of the disease. Considering the known undesirable metabolic side effect, the induction of pro-inflammatory responses and the existence of GC resistance, GCs should be used carefully. Future research should be focused not only in understanding the molecular basis of GCs side effects and resistance, but also in dissecting how GCs induce pro-inflammatory responses in order to avoid serious detrimental consequences, particularly in the brain. In the future, a combination of different therapeutic approaches may lead to a more effective treatment and may help to lower the doses or duration of GC treatment thus minimizing the risk of toxicity and drug resistance (196). Finally, taking into account inter-individual differences in patient responsiveness to GC treatment, where different molecular mechanisms might be implicated, future directions should be in support of a customized and personalized treatment to meet individual patient needs.

## Author Contributions

AL: wrote, discussed, and corrected the manuscript. MB: discussed and corrected the manuscript. CS: discussed and corrected the manuscript, performed the figures. RG: discussed and corrected the manuscript, performed the figures. AS: corrected the manuscript. EA: discussed and corrected the manuscript.

## Conflict of Interest Statement

The authors declare that there is no conflict of interest that could be perceived as prejudicing the impartiality of the research reported. The reviewer LD and handling Editor declared their shared affiliation.
